# Medicaid Primary Care Utilization and Area-Level Social Vulnerability

**DOI:** 10.1001/jamahealthforum.2025.3020

**Published:** 2025-09-05

**Authors:** Jordan Herring, Yoon Hong Park, Qian Luo, Anushree Vichare, Clese Erikson, Patricia Pittman

**Affiliations:** 1 School of Medicine, Stanford University, Stanford, California; 2 Fitzhugh Mullan Institute for Health Workforce Equity, Milken Institute School of Public Health, George Washington University, Washington, DC

## Abstract

**Importance:**

The concentration of poverty and multidimensional disadvantage has been shown to limit access to health care in these communities. There is a growing interest in using area-level socioeconomic indexes to address the unequal geographic distribution of health care resources. However, the association of area-level socioeconomic indexes with access to primary care—a key area in health policy—has not been determined.

**Objective:**

To investigate the association of Medicaid primary care utilization with the concentration of poverty and multidimensional disadvantage at the zip code level.

**Design, Settings, and Participants:**

This cross-sectional study used the 2019 Transformed-Medicaid Statistical Information System to identify variations in primary care utilization among Medicaid and the Children’s Health Insurance Program beneficiaries (age <65 years) by poverty and multidimensional disadvantage levels of their area of residence. Included beneficiaries were enrolled in Medicaid from January 1 to December 31, 2019, and were not dually eligible for Medicare. The zip code−level Social Vulnerability Index (SVI) was used to assess the likelihood of a beneficiary having an annual primary care visit, while controlling for individual beneficiary demographic and health characteristics. An activity-based approach was adopted to classify clinicians billing Medicaid for primary care and to identify primary care visits at federally qualified health centers (FQHCs). SVI results were compared with results using income-based poverty rates alone. Data analysis was performed from May 1, 2023, through February 28, 2025.

**Exposure:**

Zip code−level deciles of the SVI and poverty rates.

**Main Outcomes and Measures:**

Regression analysis was performed at the beneficiary level, using a binary indicator for having a primary care visit on a set of dummy variables for SVI deciles, controlling for age and sex interactions, disability status, and indicators for having been diagnosed with behavioral health or chronic physical health conditions.

**Results:**

The total population analyzed comprised 34 890 932 Medicaid beneficiaries (<65 years old; 54.2% female and 45.8% male), more than half of whom resided in the top 20% of socially vulnerable zip codes; approximately 33%, in the top 10%; and another 20%, in the ninth decile. Of the total, 68.1% had at least 1 primary care visit in 2019, at either a non-FQHC practice (61.1%) or a FQHC (12.7%). The probability of having a primary care visit was highest for children (age <18 years) but varied substantially by age. Compared to those residing in the first decile of the SVI (least socially vulnerable), beneficiaries in the tenth decile (most socially vulnerable) were 8.9 (95% CI, –9.9 to –7.9) percentage points (pp) less likely to have a primary care visit when not counting FQHC visits, but this increased to 4.7 (95% CI, –5.5 to –3.8) pp less likely when including FQHC visits. Beneficiaries in the tenth decile were 5.9 (95% CI, 4.9 to 6.8) pp more likely to have a FQHC visit than beneficiaries in the first decile. The SVI results identified more beneficiaries with disparities compared to the area-level poverty rate alone.

**Conclusions and Relevance:**

The findings of this cross-sectional study suggest that Medicaid policy should focus on addressing geography-based disparities in access to care using new measures to target resources. The multidimensional SVI is likely a useful tool to identify small geographic areas with barriers to accessing adequate health care. The FQHC findings suggest that substantially increasing investments and support for FQHCs would address geographic inequities in access to health care.

## Key Points

**Question:**

How is the concentration of social vulnerability in an area associated with primary care utilization by Medicaid beneficiaries?

**Findings:**

This cross-sectional study including data for 34 890 932 Medicaid beneficiaries (age <65 years) found that those residing in the most socially vulnerable areas were significantly less likely to have a primary care visit; however, the probability of having a primary care visit at a federally qualified health center for these beneficiaries increased.

**Meaning:**

These findings suggest that health workforce initiatives and outreach efforts in underserviced areas with high concentrations of social vulnerability may be more effective when they use area-level multidimensional indexes for targeted strategies to improve access to primary care.

## Teaser

This cross-sectional study assesses variations in primary care utilization associated with the concentration of multidimensional disadvantage at the zip code level among Medicaid beneficiaries.

## Introduction

Health care access and utilization in Medicaid vary substantially, both across ^1,2,3,4,5,6,7,8,9^ and within states,^10,11,12,13,14,15,16,17,18,19^ which highlights geographic inequities. Targeting areas with unmet health care needs is the cornerstone of health resource planning, but measuring true access is challenging.^20,21^ Consequently, there is a growing interest in using area-level socioeconomic indexes to target the areas in need of health care resources.^22,23,24^ Targeting geographic areas, rather than individuals, could be a more effective strategy for incentivizing competition among health plans and practices and for improving the geographic distribution of health care resources.^25^ If geographic-based socioeconomic indexes identify areas where individuals have worse access to health care, it could then make these tools useful for health care policy and planning. However, how area-level socioeconomic indexes are associated with primary care access in Medicaid—a key area in health policy for improving access to primary care—has not been determined.

Literature on concentrated poverty indicates that where an individual resides has a distinct influence on health outcomes, beyond individual factors.^26,27,28^ Measurement of poverty has now become multidimensional, demonstrating that social, economic, and environmental conditions offer a more comprehensive understanding of barriers to economic and social participation than do poverty rates based on income alone.^29,30,31^ A substantial body of research on the social determinants of health demonstrates that multidimensional disadvantage is closely associated with reduced access to health care and worse health outcomes.^32,33,34^

This study was motivated to extend the theoretical foundation of concentrated poverty^26^ to concentrated multidimensional disadvantage in the context of health care access for the most disadvantaged populations in the US. We aimed to use the Social Vulnerability Index (SVI) to investigate how primary care use among Medicaid beneficiaries varies by the concentration of multidimensional disadvantage at the zip code level.^35^ The SVI is an area-level measure of multidimensional disadvantage. Developed by the US Centers for Disease Control and Prevention,^35,36^ it has been widely used to study disparities in health care access, outcomes, and utilization.^37,38,39,40,41,42,43,44,45,46,47,48,49,50^ We focused on the probability of having a primary care visit, which is different from true access to health care,^51,52^ but may serve as a proxy for potential barriers.^20^ We also sought to assess the extent to which federally qualified health centers (FQHCs) ameliorate geography-based disparities. Established as part of an antipoverty program to improve health care access, the initial rollout of FQHCs was associated with declines in mortality rates.^53^ At FQHCs, more than 50% of patients have Medicaid coverage and more than 80% of patients whose poverty status is known are at 200% or more below the federal poverty line.^54^ In theory, primary care use at FQHCs should increase with higher levels of the SVI. In addition, to gain further insight into how concentrated multidimensional disadvantage may differ from a more parsimonious measure of concentrated poverty, we aimed to include a comparison of the SVI results with results of using only income-based poverty rates.

## Methods

This cross-sectional study was reviewed and deemed exempt by the institutional review board of George Washington University. Informed consent was not required because we used secondary data. We followed Strengthening the Reporting of Observational Studies in Epidemiology (STROBE) reporting guideline.

### Data Collection

Using the 2019 Transformed-Medicaid Statistical Information System (T-MSIS) Analytical Files (TAF), we constructed an analytical sample of Medicaid−Children’s Health Insurance Program beneficiaries (age <65 years) who were continuously enrolled in coverage from January 1 to December 31, 2019, and were not dually eligible for Medicare. We excluded those who were also enrolled in Medicare (dually enrolled) and residents of 14 states and the District of Columbia due to data quality concerns (details are available in eAppendix 1 in Supplement 1).

We used a combination of the National Plan and Provider Enumeration System and an activity-based classification developed by O’Reilly-Jacob et al^55^ to identify primary care clinicians, based on practice setting, clinical concentration, and service types for clinicians meeting an activity threshold. For clinicians not meeting the activity threshold, we defaulted to reported specialties in the National Plan and Provider Enumeration System. We classified any claim that included a primary care clinician as a primary care visit at the beneficiary level. We identified FQHC organizational National Provider Identifiers based on prior work^56^ and included all evaluation and management^57^ services from FQHCs as primary care because FQHC billing practices may differ from those of other clinic types and across states (details are available in eAppendix 2 in Supplement 1).

The SVI is an equally weighted, rank-based composite index based on variables that capture socioeconomic status, household characteristics, race and ethnicity minority status, housing type, and transportation type.^36^ The original SVI is only available at the census tract and county levels; however, the TAF includes only zip code−level data. To address the geographic mismatch, we used the 2020 data from Franchi et al^35^ who constructed a zip code−level SVI based on the original SVI methods.

### Primary Outcomes

The primary outcomes were the probability of having a primary care visit either at a non-FQHC clinic (using the individual clinician classification approach) or at a FQHC and having a primary care visit at all within the calendar year. Having at least 1 primary care visit is likely a key quality indicator for many care strategies and is likely recommended for many of the beneficiaries for preventive services. The absence of having at least 1 primary care visit at all during the year may reflect barriers with accessibility to the health care system overall.^20^

### Statistical Analysis

First, we calculated the distribution of Medicaid beneficiaries across SVI deciles using beneficiary’s residential zip codes. Then, we regressed each outcome at the individual beneficiary level on dummy variables for SVI deciles, controlling for individual demographic and health characteristics. The omitted reference decile was the first SVI decile (the least deprived areas), thus estimating the statistical difference in utilization at each SVI decile relative to the least deprived decile.

Demographic characteristics, which were self-reported in the American Community Survey, included an indicator for having a disability,^58^ and 5-year age group by sex interactions to account for variation in utilization due to both age and sex. Health characteristics included a set of indicators for having been diagnosed with behavioral health conditions (eg, depression, anxiety, bipolar disorder) and physical health chronic conditions (eg, asthma, diabetes, hypertension). eTable 2 in Supplement 1 reports the conditions used and associated diagnosis codes. Robust standard errors were clustered at the zip code level. We conducted sensitivity checks comparing the main SVI results to results from analyses that did not control for demographic and health characteristics, and to results for only US states with adequate data quality per our quality metric, which was based on the ability to observe individual clinicians on claims among the analytical sample.

To compare the main SVI results to income-based poverty rates, we repeated distribution calculation and the regression estimates but focused on a set of dummy variables corresponding to deciles based on poverty instead of SVI deciles. Data analyses were performed from Data analysis was performed from May 1, 2023, through February 28, 2025, using Stata, version 18 (Stata Corp).

## Results

The total study sample analyzed included 34 890 932 Medicaid beneficiaries (age <65 years; 54.2% female and 45.8% male). Descriptive statistics are reported in the Table. We found that 68.1% of Medicaid beneficiaries had at least 1 primary care visit at either a non-FQHC practice (61.1%) or at a FQHC (12.7%). The probability of having a primary care visit was highest for children (age <18 years) but varied substantially by age. Beneficiaries with disabilities and female beneficiaries had higher probabilities of having a primary care visit. Beneficiaries who were diagnosed with chronic conditions had much higher utilization rates overall.

**Table.** Descriptive Statistics of the Analytical Sample of 34 890 932 Medicaid or Children Health Insurance Plan Beneficiaries^a^

Characteristic	%	% With a primary care visit		
		Any	Non-FQHC	FQHC
Total (N = 34 890 932)	100.0	68.1	61.1	12.7
Sex				
Female	54.2	69.4	62.3	13.5
Male	45.8	66.4	59.6	11.8
Disability				
No disability	89.5	67.5	60.6	12.5
Has disability	10.5	72.5	65.6	14.6
Age group, y				
1-9	29.7	79.1	72.4	12.0
10-19	29.0	69.1	62.0	11.9
20-29	12.1	52.3	46.0	12.0
30-39	11.5	56.4	50.0	13.1
40-49	7.7	61.8	54.6	14.4
50-59	7.1	69.0	60.5	16.5
60-64	2.9	71.5	62.7	16.4
Diagnoses				
Behavioral health				
Anxiety	7.9	87.7	79.6	20.9
Bipolar	1.5	84.7	76.3	23.7
Depression	4.5	87.1	78.3	23.0
Schizophrenia/other psychotic disorder	1.0	77.9	68.0	22.9
Substance use disorder	3.6	79.9	71.9	22.4
Physical health				
Asthma	4.4	93.0	87.0	15.9
Chronic obstructive pulmonary disease	1.0	93.3	86.6	20.9
Diabetes	3.1	92.0	82.4	21.9
Kidney disease	0.4	91.3	86.1	18.4
Heart failure	0.2	92.4	87.4	19.6
Hypertension	4.4	93.4	83.7	21.9
Ischemic heart disease	0.2	92.7	86.9	20.8

Abbreviation: FQHC, federally qualified health center.

^a^ Data were obtained from the 2019 Transformed-Medicaid Statistical Information System Analytical Files for 36 states. The analytical sample was composed of beneficiaries from 40 states who were not dually eligible for Medicare, younger than 65 years, and enrolled in Medicaid from January 1 to December 31, 2019. Disability status was determined using Medicaid eligibility codes following MACSTATS, 2015.^58^ Non-FQHC primary care visits were identified through individual clinicians using an activity-based approach for non-FQHC settings. FQHC primary care visits were identified as all evaluation and management visits at FQHCs.

**Figure 1.**
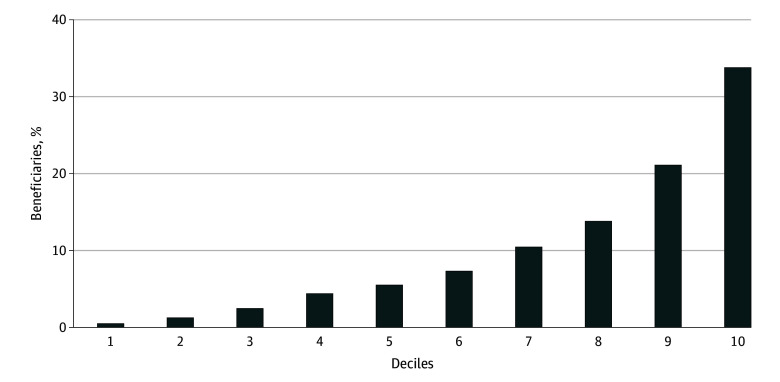
Distribution of Medicaid Beneficiaries, by Social Vulnerability Index (SVI) Deciles Data obtained from the 2019 Transformed-Medicaid Statistical Information System Analytical Files for 36 states, and 2020 SVI from Franchi et al.^35^ Beneficiaries are sorted in the SVI deciles based on zip codes. The distribution of beneficiaries across SVI deciles is reported.

Figure 1 reports the distribution of Medicaid beneficiaries across SVI deciles. We found that more than half of analytical sample of Medicaid beneficiaries resided in the top 20% of socially vulnerable zip codes; approximately 33%, in the top 10%; and another 20%, in the ninth decile.

**Figure 2.**
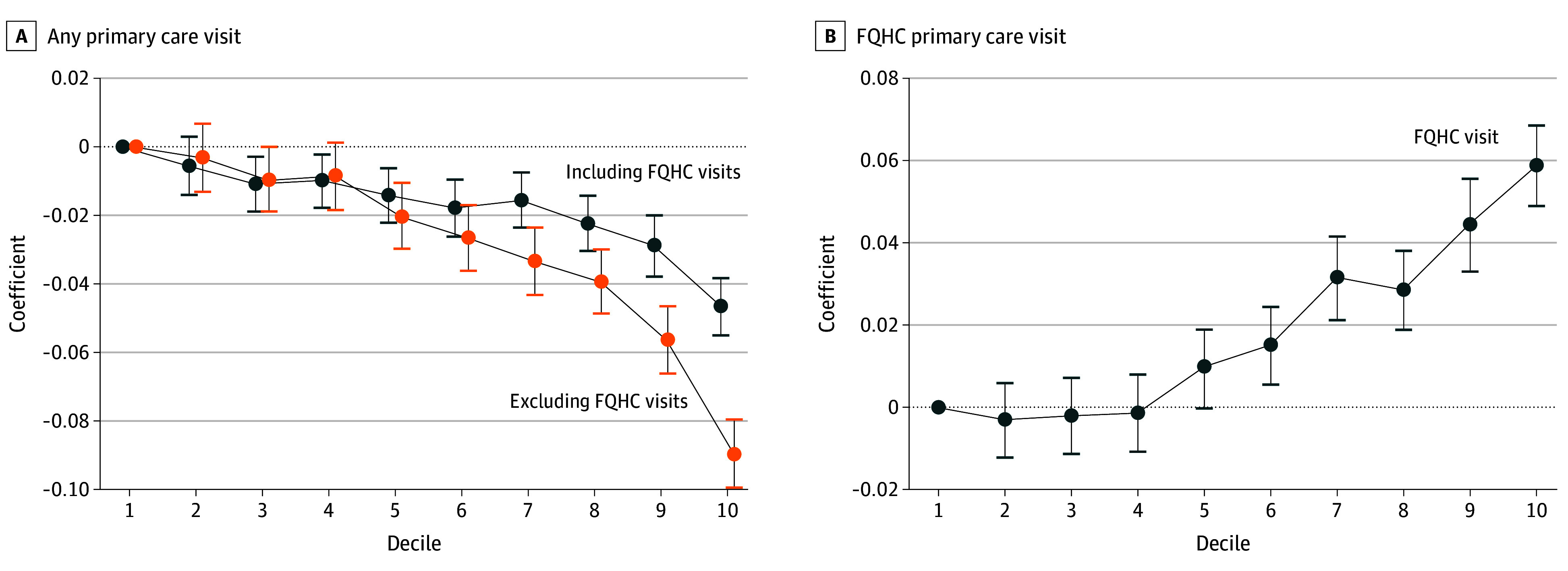
Probability of Having a Primary Care Visit, by Social Vulnerability Index (SVI) Deciles At the beneficiary level, a binary variable indicating having a primary care visit was regressed on a set of dummy variables for SVI deciles (reference category is the first decile), and the coefficients are reported with 95% CIs. The regressions include controls for 5-year age group-by-sex interactions, an indicator for having a disability, and indicators for having been diagnosed with behavioral health and physical health chronic conditions. The full regression results are reported in eTable 3 in Supplement 1. Data obtained from the 2019 Transformed-Medicaid Statistical Information System Analytical Files for 36 states, and 2020 SVI, from Franchi et al.^35^ FQHC indicates federally qualified health centers.

Figure 2A and B report the main results from the regressions controlling for individual demographic and health characteristics with 95% CIs (the full regression results are reported in eTable 3 in Supplement 1). Panel A reports the results by any primary care visit, including FQHC visits, and for only primary care visits in non-FQHC settings. Panel B reports the results for primary care visits at FQHCs only. The association between having a primary care visit and the SVI was negative overall but increased for FQHC primary care visits. Compared to beneficiaries living in the first decile of the SVI (the least socially vulnerable), beneficiaries living in the tenth decile of the SVI (the most socially vulnerable) were 8.9 (95% CI, –9.9 to –7.9) percentage points less likely to have a primary care visit when not counting FQHC visits, but this increased to 4.7 (95% CI, –5.5 to –3.8) percentage points less likely when including FQHC visits. Beneficiaries in the tenth decile were 5.9 (95% CI, 4.9 to 6.8) percentage points more likely to have a primary care visit at an FQHC than were the beneficiaries in the first decile.

Sensitivity checks comparing the main results to those without controlling for demographic and health variables and restricted to only states with adequate (low concern) data quality are reported in eFigures 2 and 3 in Supplement 1, respectively. In both cases, the probability of having a primary care visit decreased as the SVI increased. Excluding the demographic and health controls showed a smaller disparity compared to using the controls, likely associated with minor selection effects of socially vulnerable areas having a higher prevalence of chronic conditions, and thus, higher utilization rates in general.^59,60^ By adjusting for health diagnoses and accounting for these minor selection effects, a slightly larger utilization disparity emerged.

**Figure 3.**
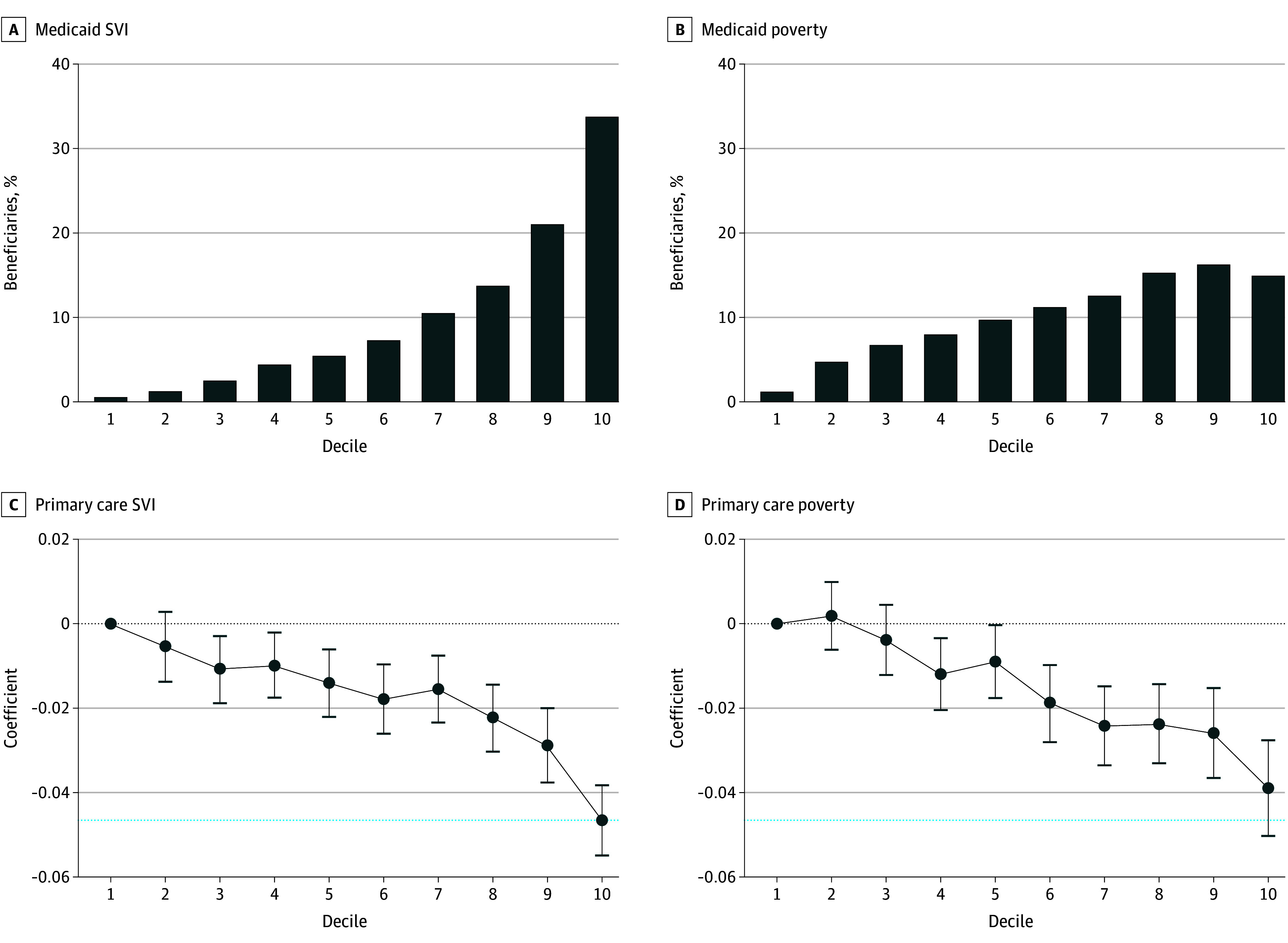
Social Vulnerability Index (SVI) Deciles Compared to Poverty Deciles A and B, Distribution of Medicaid beneficiaries by SVI vs poverty rate deciles. C and D, Probability of having a primary care visit by SVI and poverty rate deciles. Whiskers indicate with 95% CIs and the blue dashed lines denote the point estimate from the tenth decile using the SVI to compare to estimate of the tenth decile using poverty rates. Data were obtained from the 2019 Transformed-Medicaid Statistical Information System Analytical Files for 36 states, and 2020 SVI, from Franchi et al.^35^

In Figure 3 we report the distribution of beneficiaries across deciles (panel A) and the coefficients on poverty deciles for the probability of having an annual primary care visit (panel B; full regression results reported in eFigure 4 in Supplement 1). The distribution of beneficiaries across poverty deciles increased similarly to the SVI, but the level of concentration in the top deciles was lower than the SVI. The pattern across deciles based on the SVI and poverty both decreased, although the SVI estimate was slightly higher. Beneficiaries living in the tenth decile based on poverty rates were 3.9 (95% CI, –5.0 to –2.8) percentage points less likely to have a primary care visit than those in the first decile.

## Discussion

We found that Medicaid beneficiaries were disproportionately concentrated in the most socially vulnerable areas per the SVI, and those living in the most socially vulnerable areas were considerably less likely to have a primary care visit within the calendar year. Benchmarked to an overall average of 68.1% of beneficiaries having a primary care visit, those in the most socially vulnerable areas were approximately 6.9% ([4.7 ÷ 68.1] ×100) less likely to have a primary care visit, even when accounting for FQHC visits, than the average beneficiary.

These findings provide further evidence that geographic areas with a high concentration of multidimensional disadvantage potentially have worse access to health care. Previous work has found that “neighborhood effects” account for up to 15% of the geographic variation in life expectancy in the US, and places with better health outcomes tend to have higher-quality hospitals, more primary care and specialist clinicians per capita, and more frequent health care utilization,^61^ thus highlighting the importance of addressing geography-based disparities in health care access and utilization.

Many potential mechanisms may provide further insight into our study results. Neighborhood effects that diminish the accessibility of health care could be associated with a lack of investment and diminished economic growth that discourage high-quality health care clinics and hospitals from serving within these areas,^26,28^ compounding transportation barriers^62,63^ given that individuals must seek care elsewhere. Peer effects could also affect health care access, use, and outcomes.^64,65,66^ Moreover, areas where most individuals have equally poor health care access are likely to limit individuals’ abilities to exchange information on accessing high-quality health care outside of their community. Poverty stigma also operates at structural and social levels, more so than the individual level, which may discourage individuals living in high poverty areas from seeking health care.^67,68^ Racial segregation also contributes to higher rates of concentrated poverty,^69,70^ and structural racism has been associated with geographic disparities in access to health care.^11,71,72,73^

FQHCs play a critical role in serving individuals experiencing severe socioeconomic challenges,^74^ and in line with expectations, the probability of having a primary care visit at an FQHC increased considerably with area-level social vulnerability. Without FQHCs, the most socially vulnerable areas would be 8.9 percentage points less likely to have a primary care visit, but this increased to 4.7 percentage points when we included FQHC visits, reducing the disparity by more than half. However, given that only 12.7% of the overall analytical sample had a visit at an FQHC (compared to 61.1% at a non-FQHC practice), this increase was not sufficient to fully offset the geographic disparity overall and is likely due to the limited availability of FQHCs.

We found similar results in the probability of having a primary care visit using deciles of poverty rates instead of SVI deciles, although the SVI indicated a slightly larger disparity than using income-based poverty rates. Where the SVI differs more from simple poverty rates is on the distribution of Medicaid beneficiaries. For example, approximately 33% of Medicaid beneficiaries live in the top 10% of zip codes based on the SVI, but just over 15% of beneficiaries live in the top 10% of zip codes based on poverty rates. The SVI, and its multidimensionality, identifies a different set of zip codes than the poverty rate alone, capturing substantially more beneficiaries who have lower utilization rates, and potentially worse access to health care. Thus, a multidimensional disadvantage measure may increase the accuracy of identifying communities that face obstacles to accessing health care (compared to poverty rates alone).

These study results have large implications for health policy and resource targeting. The FQHC findings suggest that substantially increasing investment and support for FQHCs would directly address geographic inequities in access to health care. Most FQHCs screen for social risk factors and have many systems in place to track and address social determinants of health^75^ and employ additional staff, such as community health workers, to better meet Medicaid patients’ needs.^76,77^ However, although FQHCs have expanded considerably during the past several years, they face workforce challenges—70% of FQHCs report severe staffing shortages and recruitment shortfalls^78^—indicating more support is needed.

Because the SVI identifies communities with potentially lower access to primary care, resource planners could target the most socially vulnerable areas directly. The SVI could be further integrated into risk adjustment and value-based payment models to increase payments to health care professionals for caring for beneficiaries in socially vulnerable areas to incentivize systematic change. Similarly, the *health professional shortage areas* as defined by the US Health Resources & Services Administration could be updated to include social vulnerability as a means to proxy for lack of access to health care, and thus, incentivize practitioners and facilities to locate within these areas. For example, the SVI was recently included as a scoring factor for maternity care target areas.^22^

However, the use of multidimensional indexes in health policy has been based on using preconstructed indexes. Although we found a decline in primary care utilization as the SVI increased, other similar indexes may produce different results. For example, the Area Deprivation Index has been used to provide bonus payments to clinicians caring for beneficiaries in the most deprived areas in the Accountable Care Organization’s Realizing Equity, Access, and Community Health model^43^; however, flaws in the Area Deprivation Index construction have diminished the reliability of this particular index in improving health equity.^79^ New indexes may need to be constructed that are specific to certain policy objectives (eg, improved access, better outcomes, or addressing workforce shortages) as well as certain key drivers of disparities.^43^ Future work may also direct attention to social vulnerability within rural and/or urban stratifications.

### Limitations

Differing state administrative procedures and billing practices may limit the accuracy of quantifying national Medicaid utilization data obtained from the using the TAF. The activity-based approach may have misclassified clinicians, particularly those with smaller Medicaid patient panels or more ambiguous practice styles. Accuracy of identifying and classifying FQHC visits in the T-MSIS data are also limited because there is no comprehensive directory of FQHC billing organizations or methods to identify FQHC claims comprehensively in T-MSIS.

In addition, we captured only the variation within the population fully enrolled for the entire year. Conditioning on full enrollment may also have limited the overall evaluation of utilization given that many Medicaid beneficiaries do utilize health care despite being enrolled for only part of the year.

Moreover, the analytical sample was composed of only 36 states. This sampling bias reduces the generalizability to the national level (eg, we excluded California, which has more FQHCs than other states).

Although we used actual health care utilization as a proxy for access,^20^ utilization does not exactly translate into access. Utilization is 1 important component of accessible health care in certain communities. Additionally, we focused only on the extensive margin of primary care utilization: having any primary care visit at all. This is a narrow outcome and more detailed outcomes that identify types of visits, quality of visits, and health outcomes would provide further insights. We also focused on only 1 calendar year, and further research including multiple years would provide important information on the ability to access health care. For example, relatively healthy individuals may forgo annual wellness checks, but multiple years without primary care visits would yield further insights into access disparities.

Furthermore, we used a limited set of individual demographic factors (age, sex, disability, and health diagnoses) as controls but were not able to fully disentangle individual effects from area-level effects. For example, after controlling for other individual factors (eg, transportation access, employment, race and ethnicity), geographic variation may decrease. The TAF includes race and ethnicity variables, but there is a substantial amount of missing data across states. Additionally, we are likely underestimating diagnoses prevalence due to limitations in the T-MSIS and claims that may not comprehensively and accurately capture diagnoses.

## Conclusions

This cross-sectional study found that Medicaid beneficiaries who reside in more socially vulnerable areas are less likely to have an annual primary care visit. Medicaid policy should focus on addressing geography-based disparities in access to care using innovative new measures, such as the multidimensional SVI.

## Notes

Supplement 1. eAppendix 1. T-MSIS cleaning and data quality eAppendix 2. Activity-based primary care classification approach eAppendix 3. Identifying FQHC visits eTable 1. State-level percent of E&M office/outpatient services that have an identifiable individual provider for non-dually eligible Medicaid beneficiaries, under the age of 65, and enrolled the entire year of 2019 eFigure 1. Steps in classifying ambulatory primary care and specialist visits eTable 2. ICD-10 diagnosis codes used to identify chronic conditions eTable 3. Full regression results for SVI decile coefficients plotted in Figure 2 eFigure 2. Comparison of main SVI result to results without adjustments for demographic and health characteristics eFigure 3. Comparison of main SVI result to results for “low concern” data quality states only eFigure 4. Full regression results for poverty decile coefficients

Supplement 2. Data Sharing Statement
