# Supplementary material for: Medicaid Primary Care Utilization and Area-Level Social Vulnerability
Source: JAMA Health Forum. 2025 Sep 5;6(9):e253020. doi: 10.1001/jamahealthforum.2025.3020 (PMC12413652; doi:10.1001/jamahealthforum.2025.3020)
Supplement: Supplement 1. — eAppendix 1. T-MSIS cleaning and data quality eAppendix 2. Activity-based primary care classification approach eAppendix 3. Identifying FQHC visits eTable 1. State-level percent of E&M office/outpatient services that have an identifiable individual provider for non-dually eligible Medicaid beneficiaries, under the age of 65, and enrolled the entire year of 2019 eFigure 1. Steps in classifying ambulatory primary care and specialist visits eTable 2. ICD-10 diagnosis codes used to identify chronic conditions eTable 3. Full regression results for SVI decile coefficients plotted in Figure 2 eFigure 2. Comparison of main SVI result to results without adjustments for demographic and health characteristics eFigure 3. Comparison of main SVI result to results for “low concern” data quality states only eFigure 4. Full regression results for poverty decile coefficients [file jamahealthforum-e253020-s001.pdf]

Supplemental Online Content

Herring J, Park YH, Luo Q, Vichare A, Erikson C, Pittman P. Medicaid primary care utilization and area-level social vulnerability. *JAMA Health Forum.* Published online September 5, 2025. doi:10.1001/jamahealthforum.2025.3020

eAppendix 1. T-MSIS cleaning and data quality

eAppendix 2. Activity-based primary care classification approach

eAppendix 3. Identifying FQHC visits

eTable 1. State-level percent of E&M office/outpatient services that have an identifiable individual provider for non-dually eligible Medicaid beneficiaries, under the age of 65, and enrolled the entire year of 2019

eFigure 1. Steps in classifying ambulatory primary care and specialist visits

eTable 2. ICD-10 diagnosis codes used to identify chronic conditions

eTable 3. Full regression results for SVI decile coefficients plotted in Figure 2

eFigure 2. Comparison of main SVI result to results without adjustments for demographic and health characteristics

eFigure 3. Comparison of main SVI result to results for "low concern" data quality states only

eFigure 4. Full regression results for poverty decile coefficients

This supplemental material has been provided by the authors to give readers additional information about their work.

© 2025 Herring J et al. *JAMA Health Forum*.

eAppendix 1. T-MSIS CLEANING AND DATA QUALITY

TAF Other Services (OT) files include a blend of professional service claims, outpatient institutional claims, Medicaid capitated payments, and wrap-around payments. We are interested in the professional service claims and the institutional claims from health centers (federally qualified health centers (FQHC), rural health clinics (RHC), and community mental health centers (CMHC)). We used a combination of claim type code, bill type code, revenue center codes, and place of service codes to determine which claims are professional claims.

We started by removing claims with a claim type code of '2', '4', '5', 'B', 'D', 'E','V','X', and 'Y'. Those claims are capitated payment, service tracking claims, or supplemental payments. We then filtered out claims with valid bill type code. Bill type code is a 4-digit code reported only on UB04/CMS1450/837I (institutional) claims. We excluded any institutional claims that are not FQHC/RHC/CMHC claims (07[1/3/6/7]X). For certain states, as suggested by TAF Technical Guides, we used place of service codes (only on CMS 1500/837P professional claims) and/or revenue center codes (only on UB04/CMS1450/837I) to determine the final professional service claims to include.

Among this subset of the OT files, we then performed several correctional enhancements in order to identify the rendering providers' National Provider Identifier (NPI). For example, when the servicing provider state ID is available and the rendering provider NPI is not, we linked the state IDs with the corresponding state Annual Provider Files to fill in missing rendering NPIs.

The Centers Medicare & Medicaid Services maintains a data quality (DQ) Atlas that includes many recommendations by state for data quality metrics.[43] Based on the DQ Atlas, two

© 2025 Herring J et al. *JAMA Health Forum*.

states (AL, UT) were excluded because of the lack of usable indicators for dual enrollment with Medicare, and one other state was excluded due to high missingness of zip codes for beneficiaries (RI).

The CMS DQ Atlas reports on the availability of NPIs in the rendering provider and organizational provider fields on claims, but these quality assessments do not account for whether an individual clinician is identifiable. We created an additional data quality metric based specifically on the ability to observe individual clinicians by state. We focused on E&M office/outpatient services[44] for the metric as these services correspond to office visits where individual clinicians should be included in the claim. Among non-dually eligible Medicaid beneficiaries under the age of 65, we calculated the percent of these E&M office/outpatient services that had an individual clinician. Results are reported in appendix table A1 by state. A threshold of 80% is set based on the CMS thresholds used to identify “low quality” states per the DQ Atlas, which excludes 8 more states (DE, IL, KS, ME, MN, MT, SD, WV) and DC.

We also calculated the percent of the analytical sample that had a primary care visit by state, regardless of whether the primary care was in an FQHC or non-FQHC. We excluded 3 outliers due to implausibly low proportions of having a primary care visit less than 25% (CA, MI, and WY).

© 2025 Herring J et al. *JAMA Health Forum*.

eAppendix 2. ACTIVITY-BASED PRIMARY CARE CLASSIFICATION APPROACH

To quantify primary care and specialist utilization, we adapted an activity-based approach developed by O'Reilly-Jacob et al. to classify clinicians as either primary care or specialists to overcome limitations of workforce identification using administrative data.[45]

Administrative workforce databases may include clinician specialties that are inaccurate, out of date, or may not reflect true practice styles. Furthermore, typically all nurse practitioners (NPs) and physician assistants (PAs) are classified as primary care. But while NP and PA contributions overall to primary care has grown significantly, not all NPs and PAs practice primary care.[45,46]

Figure A1 outlines the approach. We used the thresholds for clinical concentration and atypical services proportion directly from O'Reilly-Jacob et al. The list of atypical services was obtained directly from O'Reilly-Jacob et al. We deviated from the original approach, however, by lower the activity service threshold (we set the threshold at 25 services versus 50 beneficiaries in O'Reilly-Jacob et al. to account for lower Medicaid volume per clinician), and raising the clinical concentration threshold for clinicians the care exclusively for pediatric patients. Additionally, for clinicians that did not meet the activity service threshold, we defaulted to their specialty reported in the NPPES for primary care classification. Approximately 62% of NPs and 59% of PAs meeting the outpatient service volume threshold met the activity-based criteria for primary care, increasing the accuracy of identifying which NPs and PAs practice primary care.

© 2025 Herring J et al. *JAMA Health Forum*.

eAppendix 3. IDENTIFYING FQHC VISITS

To identify evaluation & management (E&M) visits at federally qualified health centers (FQHCs), which we classified as primary care in our analysis, we used billing provider organizational national provider identifiers (NPIs) listed on the claims.[1] We identified FQHC organizational NPIs through FQHC delivery site directories from the Health Resources and Services Administration (HRSA) and the National Plan and Provider Enumeration System (NPPES). In some cases, the HRSA directories included NPIs for delivery sites, but most sites do not have an NPI reported in the HRSA directories. There is also a taxonomy code used for NPIs, reported in the NPPES, corresponding to FQHCs. However, our team found many discrepancies in that not all FQHCs have a taxonomy code in the NPPES corresponding to FQHCs (e.g., many FQHCs had a more general taxonomy code corresponding to clinics or other community-based organizations).

We matched HRSA FQHC delivery sites with organizational NPIs using fuzzy matching algorithms to match on organization name and address. In some instances, these matches were exact, and in other instances we did not find any matches. In addition to the fuzzy matching procedure, our team manually matched thousands of FQHC delivery sites to organizational NPIs in the NPPES. If we found that individual delivery sites did not have an organizational NPI, we used the grantee NPI (as one FQHC grantee can have many delivery sites).

We found that 12.7% of our analytical sample had a primary care visit at an FQHC. As a benchmark number using health center data reported by KFF, we calculated that 16.6% of

[1] Luo Q, Park H, Dor A, Moghtaderi A. Identifying National Provider Identifiers for Community Health Centers. Washington, DC: Fitzhugh Mullan Institute for Health Workforce Equity, George Washington University; 2025. https://publuu.com/flip-book/319048/1858808. Accessed 4/11/2025.

© 2025 Herring J et al. *JAMA Health Forum*.

Medicaid beneficiaries nationally were served at an FQHC.[2] The dissonance between our 12.7% and KFF's 16.6% may be due to a variety of factors, such as: our analysis excluded 13 states (e.g., such as California, where there are many FQHCs, thus our analytical sample may have lower service at FQHCs), the KFF figure of 16.6% includes all services and is not specific to primary care or office-based medical care (e.g., may include dental visits, mental health-only visits, etc.) and thus the proportion of Medicaid beneficiaries with a primary care visit at an FQHC is likely to be a subset of the broader group reported by KFF, and the KFF report may capture duplicate counting of patients as each health center/grantee reports the number of patients served separately and if patients are served at multiple centers, then they may be counted multiple times (whereas our analysis is based on unduplicated patient counts).

[2] KFF analysis on FQHC patients: https://www.kff.org/medicaid/issue-brief/community-health-center-patients-financing-and-services/. Accessed 4/11/2025.
KFF data on total Medicaid enrollment: https://www.kff.org/medicaid/state-indicator/distribution-of-medicaid-enrollees-by-enrollment-group/?currentTimeframe=2&sortModel=%7B%22colId%22:%22Location%22,%22sort%22:%22asc%22%7D. Accessed 4/11/2025.

© 2025 Herring J et al. *JAMA Health Forum*.

eTable 1. State level percent of E&M office/outpatient services that have an identifiable individual provider for non-dually eligible Medicaid beneficiaries, under the age of 65, and enrolled the entire year of 2019

Excluded States (< 80%)		Included States – Medium Concern (80-90%)		Included States – Low Concern (≥ 90%)	
**State**	**%**	**State**	**%**	**State**	**%**
MN	0.2	IA	80.5	SC	90.9
DE	0.6	DC	80.6	NH	91.5
SD	56.5	MD	81.4	NJ	92.4
WV	64.6	VA	82.5	AK	92.6
IL	70.2	NM	84.8	NY	92.9
KS	70.7	PA	86.2	RI	93.7
MT	71.8	ND	86.5	AZ	93.8
ME	72.8	CA	87.2	MS	94.6
		TX	87.9	KY	94.6
		MI	88.0	PR	95.5
		NE	88.5	FL	95.6
		MA	88.7	OH	96.5
		GA	89.7	NV	97.1
				LA	97.1
				MO	97.2
				WI	97.6
				HI	97.8
				ID	98.3
				CT	98.3
				OR	98.5
				TN	98.5
				WY	98.5
				AR	98.8
				WA	98.9
				IN	99.5
				CO	99.6
				VI	99.8
				VT	99.8
				OK	99.9
				NC	99.9

SOURCE 2019 TAF.

© 2025 Herring J et al. *JAMA Health Forum*.

eFigure 1: Steps in classifying ambulatory primary care and specialist visits

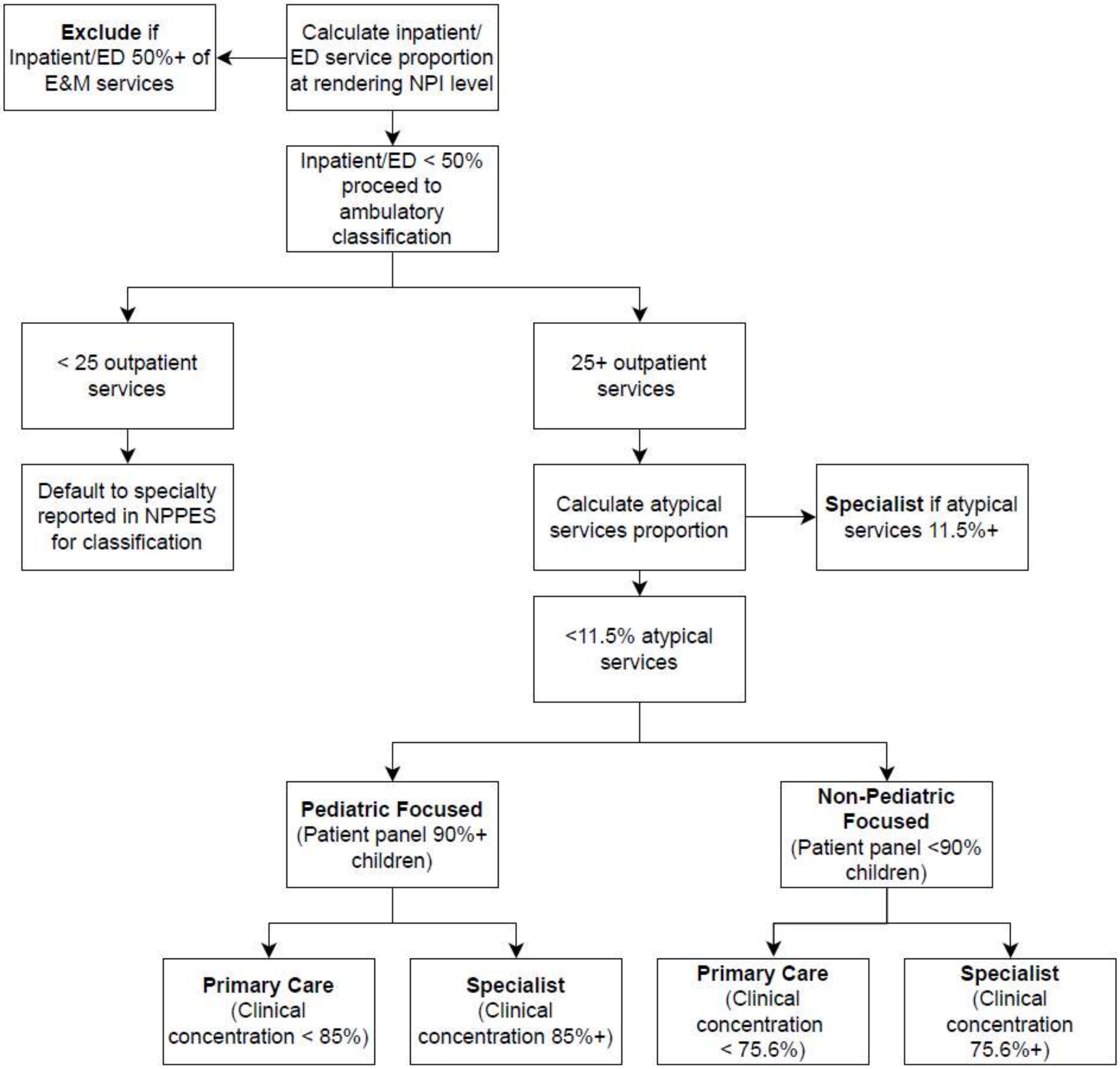

NOTES The above activity-based approach is adapted from the original approach used in O'Reilly-Jacob et al.

© 2025 Herring J et al. *JAMA Health Forum*.

eTable 2. ICD-10 diagnosis codes used to identify chronic conditions

Condition	Associated ICD-10 Codes	Source
**Behavioral Health**		
Anxiety, dissociative, stress-related, somatoform and other nonpsychotic mental disorders	F40-F48 (F4*)	ICD10Data
Bipolar	F31	ICD10Data
Depression	F32, F33	ICD10Data
Schizophrenia, schizotypal, delusional, and other non-mood psychotic disorders	F20-F29 (F2*)	ICD10Data
Substance Use Disorder (SUD)	F10-F19 (F1*)<br>T407, T408, T409, T410, T400, T401, T402, T403, T404, T406, T426, T427, T405, T436, F10, G312, G621, I426, K292, K70, O354	ICD10Data;<br>MDC 20 Alcohol/Drug Use & Alcohol/Drug Induced Organic Mental Disorders<br>Assignment of Diagnosis Codes;<br>Peterson et al. (2021).
**Physical Health**		
Asthma	J45	MDC 4 Diseases & Disorders of the Respiratory System<br>Bronchitis and Asthma
Kidney Failure & Chronic Kidney Disease	E883, I120, I129, I131, N170, N171, N178, N179, N181, N182, N184, N185, N186, N189, N19, R34	MDC 11 Diseases & Disorders of the Kidney & Urinary Tract
Chronic Obstructive Pulmonary Disease (COPD)	J411, J418, J42, J430, J431, J432, J438, J439, J440, J441, J449, J470, J471, J479, J684, J688, J689, Q334	MDC 4 Diseases & Disorders of the Respiratory System<br>Chronic Obstructive Pulmonary Disease
Diabetes	E08, E09, E10, E11, E12, E13	MDC 10 Endocrine, Nutritional & Metabolic Diseases & Disorders<br>Diabetes; ICD10Data
Heart Failure	I508, I509, R570, R579	MDC 05 Diseases & Disorders of the Circulatory System
Hypertension	I10, I119, I150, I151, I152, I158, I159, I160, I161, I169, I1A0, N262	MDC 05 Diseases and disorders of the circulatory system<br>Hypertension
Ischemic Heart Disease	I200, I201, I208, I209, I240, I248, I249	MDC 05 Diseases & Disorders of the Circulatory System<br>Angina Pectoris

© 2025 Herring J et al. *JAMA Health Forum*.

eTable 3: Full regression results for SVI decile coefficients plotted in figure 2

	(1) Had any primary care visit (incl. CHC)		(2) Had any primary care visit (excl. CHC)		(3) Had CHC visit	
	Coeff.	SE	Coeff.	SE	Coeff.	SE
**SVI Decile (Ref.: 1st decile)**						
2	-0.00550	(0.00428)	-0.00307	(0.00503)	-0.00308	(0.00454)
3	-0.0109**	(0.00406)	-0.00951*	(0.00484)	-0.00206	(0.00471)
4	-0.00988*	(0.00399)	-0.00855	(0.00496)	-0.00143	(0.00476)
5	-0.0141***	(0.00408)	-0.0201***	(0.00487)	0.00933	(0.00485)
6	-0.0179***	(0.00422)	-0.0264***	(0.00490)	0.0149**	(0.00480)
7	-0.0156***	(0.00407)	-0.0333***	(0.00496)	0.0314***	(0.00513)
8	-0.0224***	(0.00408)	-0.0393***	(0.00475)	0.0284***	(0.00487)
9	-0.0288***	(0.00455)	-0.0563***	(0.00495)	0.0443***	(0.00568)
10	-0.0466***	(0.00424)	-0.0894***	(0.00508)	0.0587***	(0.00497)
**Age-by-Sex Interactions (Ref.: Age 0–5)**						
Age 5–9 x F	-0.100***	(0.000675)	-0.102***	(0.000717)	-0.00572***	(0.000638)
Age 5–9 x M	-0.105***	(0.000666)	-0.107***	(0.000716)	-0.00525***	(0.000619)
Age 10–14 x F	-0.145***	(0.000789)	-0.150***	(0.000871)	-0.00920***	(0.000778)
Age 10–14 x M	-0.149***	(0.000802)	-0.155***	(0.000869)	-0.00880***	(0.000778)
Age 15–19 x F	-0.180***	(0.000976)	-0.184***	(0.00104)	-0.00197*	(0.000974)
Age 15–19 x M	-0.244***	(0.00101)	-0.242***	(0.00109)	-0.0245***	(0.000988)
Age 20–24 x F	-0.311***	(0.00217)	-0.310***	(0.00219)	-0.000613	(0.00146)
Age 20–24 x M	-0.452***	(0.00216)	-0.436***	(0.00226)	-0.0480***	(0.00153)
Age 25–29 x F	-0.314***	(0.00208)	-0.313***	(0.00217)	-0.000451	(0.00157)
Age 25–29 x M	-0.441***	(0.00203)	-0.431***	(0.00224)	-0.0382***	(0.00167)
Age 30–34 x F	-0.310***	(0.00201)	-0.309***	(0.00213)	-0.00313*	(0.00146)
Age 30–34 x M	-0.432***	(0.00211)	-0.424***	(0.00237)	-0.0351***	(0.00162)
Age 35–39 x F	-0.305***	(0.00199)	-0.304***	(0.00211)	-0.00445**	(0.00146)
Age 35–39 x M	-0.417***	(0.00215)	-0.412***	(0.00239)	-0.0298***	(0.00165)
Age 40–44 x F	-0.298***	(0.00210)	-0.298***	(0.00221)	-0.00334*	(0.00154)
Age 40–44 x M	-0.398***	(0.00225)	-0.397***	(0.00249)	-0.0245***	(0.00169)
Age 45–49 x F	-0.287***	(0.00222)	-0.291***	(0.00242)	0.00136	(0.00169)
Age 45–49 x M	-0.379***	(0.00228)	-0.383***	(0.00265)	-0.0170***	(0.00179)
Age 50–54 x F	-0.283***	(0.00219)	-0.292***	(0.00252)	0.00671***	(0.00186)
Age 50–54 x M	-0.361***	(0.00215)	-0.373***	(0.00257)	-0.00736***	(0.00194)
Age 55–59 x F	-0.286***	(0.00219)	-0.300***	(0.00262)	0.00603**	(0.00195)
Age 55–59 x M	-0.351***	(0.00210)	-0.369***	(0.00259)	-0.00158	(0.00204)
Age 60–64 x F	-0.287***	(0.00223)	-0.303***	(0.00264)	0.00289	(0.00205)
Age 60–64 x M	-0.345***	(0.00213)	-0.364***	(0.00264)	-0.00262	(0.00210)
**Disabled**	0.0319***	(0.00119)	0.0414***	(0.00133)	-0.00952***	(0.00140)
**Ever Diagnosed With**						
Anxiety	0.184***	(0.000779)	0.173***	(0.000921)	0.0686***	(0.00124)
Bipolar	0.0983***	(0.00126)	0.0911***	(0.00120)	0.0544***	(0.00169)
Depression	0.148***	(0.000932)	0.134***	(0.000919)	0.0666***	(0.00131)
Schizophrenia	0.0637***	(0.00128)	0.0414***	(0.00132)	0.0573***	(0.00182)
SUD	0.146***	(0.00167)	0.139***	(0.00159)	0.0686***	(0.00188)
Asthma	0.204***	(0.000987)	0.217***	(0.00104)	0.0236***	(0.00118)
COPD	0.129***	(0.000982)	0.147***	(0.00118)	0.0191***	(0.00141)
Diabetes	0.230***	(0.000876)	0.212***	(0.000929)	0.0609***	(0.00110)
Kidney Disease	0.0847***	(0.00106)	0.118***	(0.00134)	-0.00885***	(0.00133)
Heart Failure	0.0545***	(0.00153)	0.0869***	(0.00181)	-0.00439*	(0.00190)
Hypertension	0.276***	(0.00124)	0.256***	(0.00124)	0.0611***	(0.00155)
IHD	0.0890***	(0.00204)	0.118***	(0.00240)	0.00817**	(0.00299)
N	34,890,932		34,890,932		34,890,932	

SOURCE 2019 TAF for 36 states; 2020 SVI from Franchi et al. (2024).

© 2025 Herring J et al. *JAMA Health Forum*.

eFigure 2. Comparison of main SVI result to results without adjustments for demographic and health characteristics

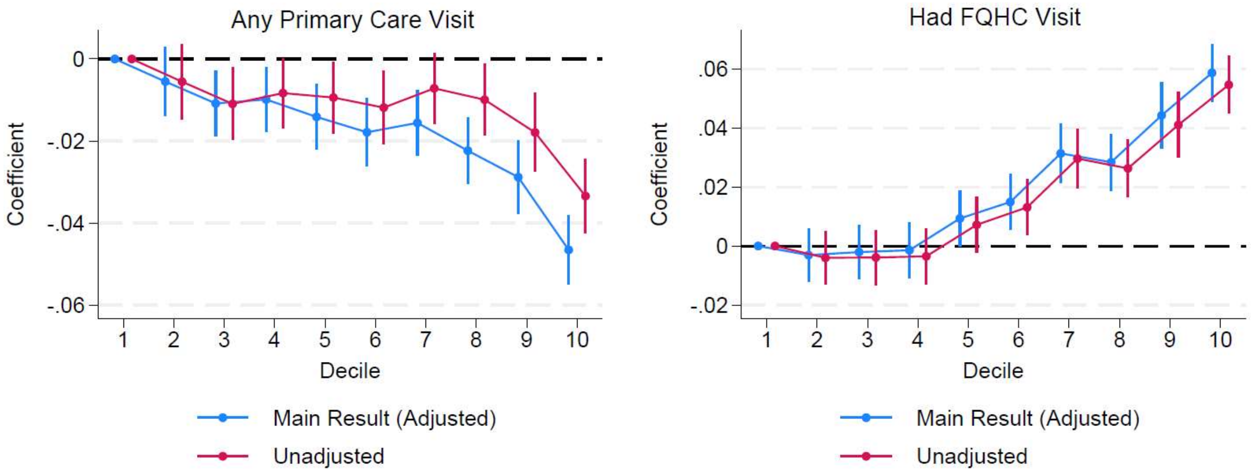

SOURCE 2019 TAF for 36 states; 2020 SVI from Franchi et al. (2024).

© 2025 Herring J et al. *JAMA Health Forum*.

eFigure 3: Comparison of main SVI result to results for "low concern" data quality states only

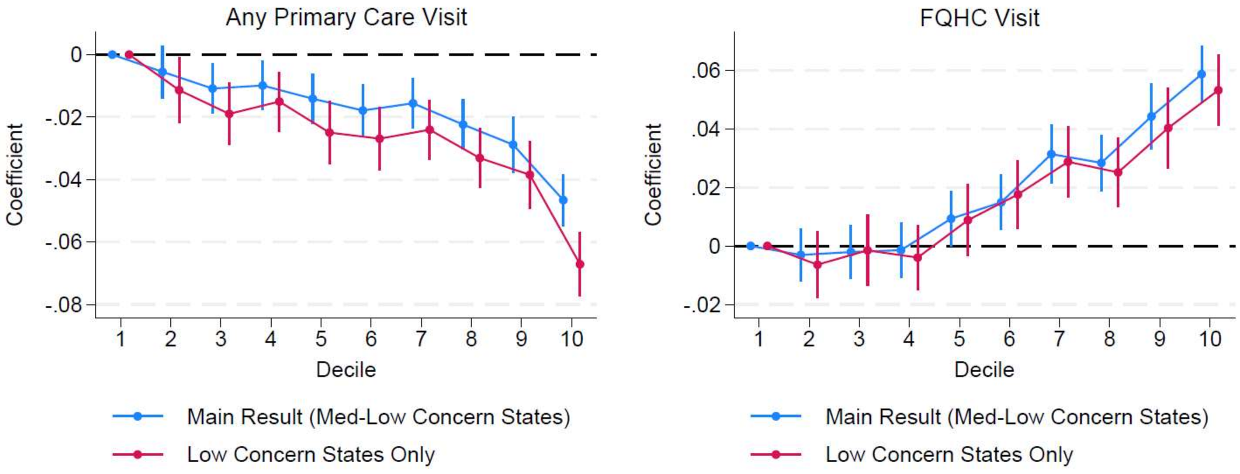

SOURCE 2019 TAF for 36 states; 2020 SVI from Franchi et al. (2024).

© 2025 Herring J et al. *JAMA Health Forum*.

eFigure 4: Full regression results for poverty decile coefficients

	(1)	(2)	(3)	(4)
	SVI Deciles		Poverty Declines	
	Coeff.	SE	Coeff.	SE
**Decile (Ref.: 1st decile)**				
2	-0.00550	(0.00428)	0.00182	(0.00410)
3	-0.0109**	(0.00406)	-0.00388	(0.00431)
4	-0.00988*	(0.00399)	-0.0121**	(0.00433)
5	-0.0141***	(0.00408)	-0.00904*	(0.00448)
6	-0.0179***	(0.00422)	-0.0188***	(0.00467)
7	-0.0156***	(0.00407)	-0.0242***	(0.00481)
8	-0.0224***	(0.00408)	-0.0238***	(0.00475)
9	-0.0288***	(0.00455)	-0.0259***	(0.00545)
10	-0.0466***	(0.00424)	-0.0389***	(0.00576)
**Age-by-Sex Interactions (Ref.: Age 0–5)**				
Age 5–9 x F	-0.100***	(0.000675)	-0.100***	(0.000672)
Age 5–9 x M	-0.105***	(0.000666)	-0.105***	(0.000663)
Age 10–14 x F	-0.145***	(0.000789)	-0.146***	(0.000786)
Age 10–14 x M	-0.149***	(0.000802)	-0.149***	(0.000800)
Age 15–19 x F	-0.180***	(0.000976)	-0.180***	(0.000973)
Age 15–19 x M	-0.244***	(0.00101)	-0.244***	(0.00100)
Age 20–24 x F	-0.311***	(0.00217)	-0.310***	(0.00216)
Age 20–24 x M	-0.452***	(0.00216)	-0.452***	(0.00214)
Age 25–29 x F	-0.314***	(0.00208)	-0.313***	(0.00207)
Age 25–29 x M	-0.441***	(0.00203)	-0.441***	(0.00201)
Age 30–34 x F	-0.310***	(0.00201)	-0.309***	(0.00200)
Age 30–34 x M	-0.432***	(0.00211)	-0.432***	(0.00208)
Age 35–39 x F	-0.305***	(0.00199)	-0.305***	(0.00198)
Age 35–39 x M	-0.417***	(0.00215)	-0.416***	(0.00213)
Age 40–44 x F	-0.298***	(0.00210)	-0.298***	(0.00209)
Age 40–44 x M	-0.398***	(0.00225)	-0.398***	(0.00222)
Age 45–49 x F	-0.287***	(0.00222)	-0.286***	(0.00219)
Age 45–49 x M	-0.379***	(0.00228)	-0.378***	(0.00224)
Age 50–54 x F	-0.283***	(0.00219)	-0.283***	(0.00216)
Age 50–54 x M	-0.361***	(0.00215)	-0.361***	(0.00211)
Age 55–59 x F	-0.286***	(0.00219)	-0.286***	(0.00215)
Age 55–59 x M	-0.351***	(0.00210)	-0.350***	(0.00206)
Age 60–64 x F	-0.287***	(0.00223)	-0.287***	(0.00219)
Age 60–64 x M	-0.345***	(0.00213)	-0.344***	(0.00209)
**Disabled**	0.0319***	(0.00119)	0.0321***	(0.00121)
**Ever Diagnosed With**				
Anxiety	0.184***	(0.000779)	0.185***	(0.000801)
Bipolar	0.0983***	(0.00126)	0.0986***	(0.00127)
Depression	0.148***	(0.000932)	0.149***	(0.000943)
Schizophrenia	0.0637***	(0.00128)	0.0632***	(0.00130)
SUD	0.146***	(0.00167)	0.147***	(0.00169)
Asthma	0.204***	(0.000987)	0.204***	(0.000987)
COPD	0.129***	(0.000982)	0.131***	(0.00100)
Diabetes	0.230***	(0.000876)	0.230***	(0.000872)
Kidney Disease	0.0847***	(0.00106)	0.0843***	(0.00106)
Heart Failure	0.0545***	(0.00153)	0.0541***	(0.00153)
Hypertension	0.276***	(0.00124)	0.275***	(0.00125)
IHD	0.0890***	(0.00204)	0.0887***	(0.00204)
N	34,890,932		34,901,208	

SOURCE 2019 TAF for 36 states; 2020 SVI from Franchi et al. (2024).

© 2025 Herring J et al. *JAMA Health Forum*.
