# Supplementary material for: Medicaid Primary Care Utilization and Area-Level Social Vulnerability
Source: JAMA Health Forum. 2025 Sep 5;6(9):e253020. doi: 10.1001/jamahealthforum.2025.3020 (PMC12413652; doi:10.1001/jamahealthforum.2025.3020)
Supplement: Supplement 2. — Data Sharing Statement [file jamahealthforum-e253020-s002.pdf]

Data Sharing Statement

Herring. Medicaid Primary Care Utilization and Area-Level Social Vulnerability. *JAMA Health Forum*. Published September 05, 2025. doi:10.1001/jamahealthforum.2025.3020

Data
Data available: No

Additional Information
Explanation for why data not available: Medicaid data is subject to DUA.
